# Supplementary material for: COZOID: contact zone identifier for visual analysis of protein-protein interactions
Source: BMC Bioinformatics. 2018 Apr 6;19:125. doi: 10.1186/s12859-018-2113-6 (PMC5889581; doi:10.1186/s12859-018-2113-6)
Supplement: Supplementary file 2 — Software build. Executable binary file of the software tool. (ZIP 118,784 kb) [file 12859_2018_2113_MOESM2_ESM.zip › cozoid/java/docs/beansbinding-1.2.1-doc.zip!/org/jdesktop/observablecollections/class-use/ObservableMap.html]

Uses of Interface org.jdesktop.observablecollections.ObservableMap (Beans Binding)


---


|  |  |  |
| --- | --- | --- |
| **Overview** \| **Package** \| **Class** \| **Use** \| **Tree** \| **Deprecated** \| **Index** \| **Help** | |  |
| PREV   NEXT | **FRAMES**    **NO FRAMES**     **All Classes** |  |


---


## **Uses of Interface org.jdesktop.observablecollections.ObservableMap**

| Packages that use ObservableMap | |
| --- | --- |
| **org.jdesktop.observablecollections** | Provides support for observing changes to collection classes. |

Uses of ObservableMap in org.jdesktop.observablecollections

Methods in org.jdesktop.observablecollections that return ObservableMap

`static

<K,V> ObservableMap<K,V>`

`ObservableCollections.observableMap(java.util.Map<K,V> map)`
  
          Creates and returns an `ObservableMap` wrapping the supplied
`Map`.

| Methods in org.jdesktop.observablecollections with parameters of type ObservableMap | |
| --- | --- |
| `void` | `ObservableMapListener.mapKeyAdded(ObservableMap map, java.lang.Object key)`             Notification that a key has been added. |
| `void` | `ObservableMapListener.mapKeyRemoved(ObservableMap map, java.lang.Object key, java.lang.Object value)`             Notification that a key has been removed |
| `void` | `ObservableMapListener.mapKeyValueChanged(ObservableMap map, java.lang.Object key, java.lang.Object lastValue)`             Notification that the value of an existing key has changed. |

---


|  |  |  |
| --- | --- | --- |
| **Overview** \| **Package** \| **Class** \| **Use** \| **Tree** \| **Deprecated** \| **Index** \| **Help** | |  |
| PREV   NEXT | **FRAMES**    **NO FRAMES**     **All Classes** |  |


---
